# Engineered Bioluminescent Indicator Enables the Brain Imaging of Kinase Inhibitors

**DOI:** 10.1021/acscentsci.3c00367

**Published:** 2023-04-11

**Authors:** Zhao Ma, Minyong Li

**Affiliations:** Department of Medicinal Chemistry, Key Laboratory of Chemical Biology (MOE), School of Pharmaceutical Sciences, Cheeloo College of Medicine, Shandong University, Jinan, Shandong 250012, China

With the emergence of an aging society, brain diseases such as brain tumors, Alzheimer’s disease (AD), Parkinson’s disease (PD), and stroke account for an increasing proportion of global diseases, resulting in a growing demand for therapeutic drugs.^1^ Due to the high failure rate of up to 92%, drug development for brain diseases remains a formidable challenge.^2^ A major difficulty in brain drug development is the presence of the blood-brain barrier (BBB), which serves as a “gatekeeper” of the brain to precisely control the entry and exit of substances.^3^ Since the BBB blocks almost 100% of macromolecular drugs and more than 98% of small-molecule drugs, resulting in a significantly lower drug concentration in cerebrospinal fluid than in plasma, the conventional plasma drug concentration–time curve cannot reflect the real behavior of drugs in the brain. To understand the difference in drug concentration between the blood and brain, significant efforts have been invested to harvest and process brain tissues, which is a time-consuming and costly endeavor. Besides the BBB permeability, a more important index of the success of a drug to treat brain diseases is its actual biochemical and physiologic effects produced in the brain. Monitoring these drug activities in real-time is highly challenging.

**Figure 1:**
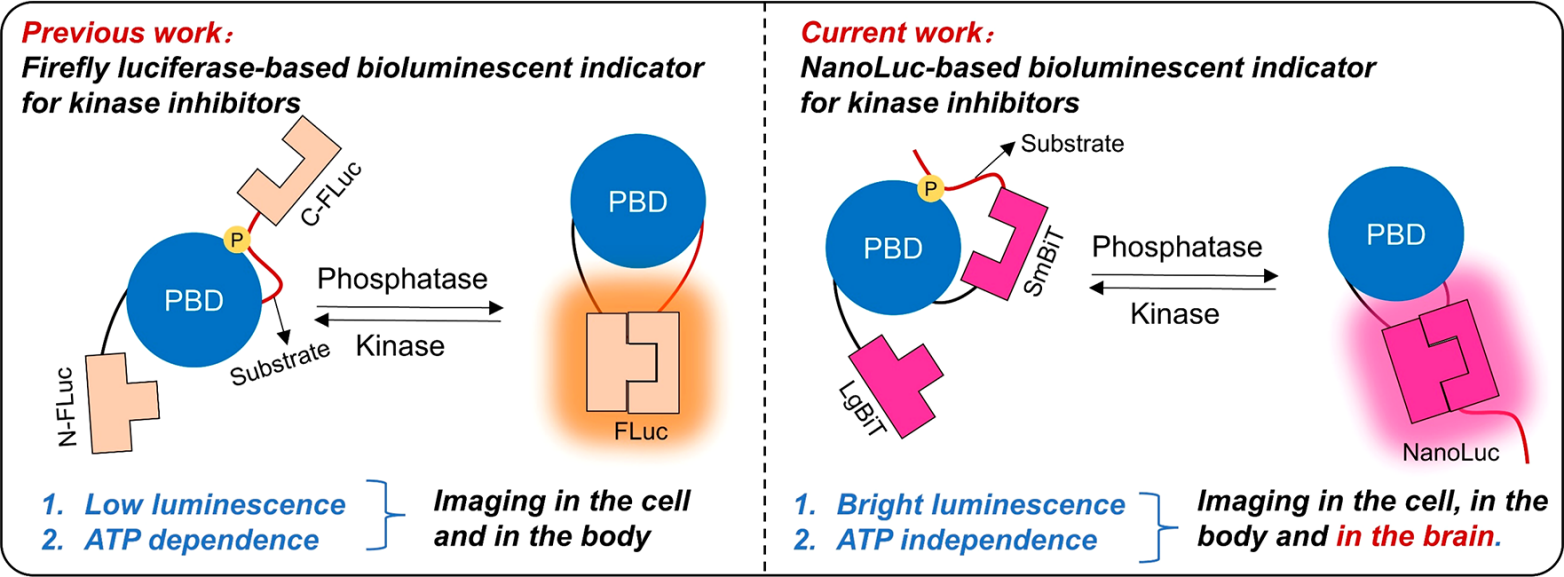
Proposed mechanism and applications of firefly luciferase-based bioluminescent indicators developed in the past (left) and a NanoLuc-based bioluminescent indicator constructed currently (right).

Visualization-based techniques are a useful means to monitor and analyze drugs in the brain, for example, an isotopically labeled drug makes it possible to measure its concentrations in the brain with the assistance of a PET or SPECT scanner,^4^ and the mass spectrometry imaging (MSI) technique enables the compound-specific imaging of drug distribution in brain tissue.^5^ This type of method greatly contributes to the determination of drug penetration into the brain, but little to the elaboration of a drug’s activity. As a powerful visualization platform, the bioluminescent reporter system shows considerable potential in the analysis and screening of drug activities. A variety of such systems have been well established based on firefly luciferase for different classes of drug targets, such as transcription factor, nuclear receptor, and kinase (Figure 1).^6^ However, its application in investigating drugs or targets in the brain has not yet been achieved. Construction of a robust bioluminescent reporter in the brain represents a promising strategy to investigate the BBB permeability and activities of a candidate brain drug simultaneously, thus greatly facilitating drug development for treating brain diseases.

In this issue of *ACS Central Science*, Su, Lin, and co-workers develop kinase-modulated bioluminescent indicators (KiMBIs) for the real-time imaging of drug behaviors in the brain by engineering a modern luciferase NanoLuc (Figure 1).^1^ Compared to that of conventional firefly luciferase-based bioluminescent reporters developed in the past, the signals of KiMBIs are more robust because the function of NanoLuc is ATP independence.^7^ Moreover, an optimized NanoLuc substrate was adopted to guarantee brighter imaging signals in the brain.^8^ As a proof of concept, the authors first constructed PKA KiMBIs with a topology LgBiT-FHA (Forkhead-associated)-linker-substrate-SmBiT, in which the linker needs optimization to allow the phosphorylated substrate to reach its binding site on FHA, and verified that the PKA KiMBIs emit weaker bioluminescence upon PKA activation. Considering the popularity of drugs targeting the Ras-ERK pathway, the authors then focused on the development of a KiMBI for EKR inhibition. After optimizing the topology structures, the bKiMBI was screened as it displayed a >10-fold bioluminescence response to the EKR inhibition by Vx-11e. It was further validated that bKiMBI could be specifically activated by various MEK or ERK inhibitors in cancer cells. To improve tissue penetration, the fusion of a fluorescent protein of long-wave emission into bKiMBI generated oKiMBI and tKiMBI, and the latter was chosen for studies in vivo.

The authors adopted the subcutaneous tumor xenograft model to confirm the functions of tKiMBI on imaging of ERK inhibition. By expressing KiMBI in the brain, the authors verified that tKiMBI is a useful tool for the evaluation of BBB permeability of kinase inhibitors and noninvasive characterization of their activities in the brain. Given its comprehensive advantages to address the challenges in drug development for treating brain diseases, we believe that the KiMBI technology has a bright future in bringing effective drugs to patients.
